# Optimizing Salt Lipophilicity for Reference Electrodes Based on Organic Electrolytes

**DOI:** 10.1021/acsmeasuresciau.6c00145

**Published:** 2026-08-03

**Authors:** Nikolai Yu. Tiuftiakov, Supakron Kittikomoldej, Eric Bakker

**Affiliations:** † Department of Inorganic and Analytical Chemistry, University of Geneva, Quai Ernest-Ansermet 30, CH-1211 Geneva, Switzerland; ‡ Department of Chemistry and Center of Excellence for Innovation in Chemistry, Faculty of Science, Mahidol University, Rama 6 Road, Bangkok 10400, Thailand

**Keywords:** Reference electrodes, organic electrolytes, potentiometry, potentiometric titration, organic salt lipophilicity

## Abstract

Reference electrodes (REs) based on membranes doped with organic electrolytes offer an attractive route toward versatile, miniaturized, and maintenance-free sensing platforms. Nevertheless, the choice of the reference electrolyte remains a critical design parameter that determines the performance of the corresponding REs. Different ranges of ionic liquid and solid organic salt lipophilicity have previously been explored for RE design, each characterized by different intrinsic limitations. Although the increased partitioning of less lipophilic electrolytes from the reference membrane to the sample establishes stable RE potentials, it also results in rapid electrolyte loss and sample contamination. This might in principle be mitigated with highly lipophilic organic electrolytes, but even minor deviations from the ideal stoichiometric composition of such salts induce parasitic responses of the RE to sample species. To combine the advantages of both characteristics, we investigate here the intermediate electrolyte lipophilicity range that has largely remained underexplored for RE design. Ionic liquids with lipophilicities exceeding that of commercial analogues based on the bis­(trifluoromethanesulfonyl)­imide ([C_1_C_1_N^–^]) anion were prepared via potentiometric titrations by systematically varying the lipophilicity of the tetraalkylammonium used as countercation. The best-performing composition, based on tetrahexylammonium ([THA^+^]­[C_1_C_1_N^–^]), provided reproducible and stable reference electrode potentials in both bulk solutions and under flow conditions, giving a potential variation of only 0.16 mV for solutions bracketing the physiological electrolyte range. This stability was accompanied by dramatically reduced leaching of the salt from the membrane, which was confirmed via quartz crystal microbalance measurements and the absence of chemical poisoning of ion-selective membranes placed in the same solution.

## Graphical abstract

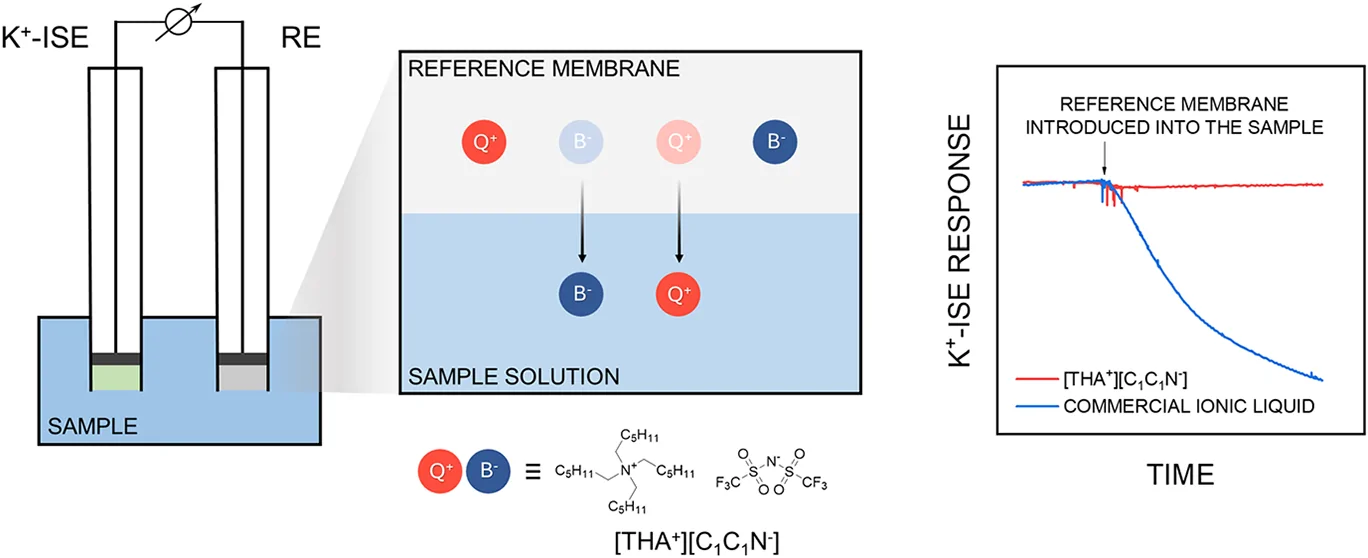

## Introduction

The continued advancement of ion-selective electrodes (ISEs) has provided robust long-term stable tools for the analysis of a wide range of analytes.^1^ They can be readily miniaturized and tailored to specific analytical applications, including rapidly developing wearable sensing technologies^2,3^ or *on-site* and *in situ* environmental monitoring.^4^ To keep up with this progress, increasingly stringent requirements have also been placed on the design of reference electrodes (REs). Conventional double-junction reference electrodes, although ubiquitous for more than a century, are challenging to miniaturize^5^ and suffer from mutual contamination of the bridge electrolyte and the sample.^6,7^ Liquid junction-free REs of various kinds have been proposed to address these limitations.^5,8^ Among them, REs based on reference membranes doped with organic electrolytes have emerged as particularly promising due to their compatibility with a wide range of materials previously developed for ISEs and overall versatility of their design,^5,9^ which can easily be further optimized for wearable^10,11^ or clinical applications.^12,13^

Ionic liquids (ILs) have been the organic electrolytes of choice for REs^5^ ever since the initial studies by Kakiuchi.^14^ They can be used in combination with various membrane materials and supports, while their moderate lipophilicity allows to establish an equilibrium distribution between the reference membrane and its aqueous vicinity,^15^ thereby rendering the corresponding interfacial potential stable and independent of the sample composition. Unfortunately, this moderate ionic liquid lipophilicity presents an inherent trade-off: while higher partitioning indeed suppresses the influence of the sample species on the electrode potential, it also promotes the electrolyte loss into the sample solution, which can significantly limit the electrode lifetime and result in sample contamination. This behavior is consistent with the relatively low partitioning constants of the commonly used commercial ILs^16^ and, in extreme cases, may result in poisoning of polymeric membrane ISEs, thus making IL-based REs incompatible with small sample volumes.^17^ Buhlmann and Mousavi demonstrated across several studies that IL-based REs can nevertheless be successfully integrated into compact paper- and yarn-based devices and applied to the analysis of biological fluids such as serum, urine, saliva and milk.^10,18–21^ These approaches rely on device-level strategies, such as minimizing the exposed area of the reference electrode relative to the volume of the used sample. However, while such strategies enable practical measurements, they only mitigate the consequences of reference electrolyte transfer into the sample rather than address the core issue itself.

Electrolyte loss and sample contamination could, in principle, be minimized by significantly increasing the lipophilicity of the used organic electrolyte. However, this strategy must be approached with caution due to the growing influence of electrolyte purity. As demonstrated by Lindner et al.,^22^ minute excess of one highly lipophilic ion over the other may essentially act as an ion-exchanger, promoting the transfer of the sample species of the opposite charge into the membrane and giving rise to a Nernstian response toward interferences. We later showed that this effect remains significant even at ion mismatch of 0.1%, making highly lipophilic electrolytes alone essentially unsuitable for use in reference membranes.^23^ In a follow-up study, we proposed a seemingly paradoxical approach combining mismatched highly lipophilic electrolytes with ionophores for RE design.^24^ In this case, the presence of an ionophore leverages the ionic mismatch and changes the potential stabilization mechanism from simple partitioning of the organic electrolyte to ion-exchange between the deficient organic ion and the ion complexed by the ionophore. This strategy requires thorough optimization of the relationship between the electrolyte lipophilicity and the ion–ionophore complex stability and necessitates the presence of the target ion of the ionophore in the sample, which may limit its practical applicability.

An intermediate lipophilicity range may present a “sweet spot” between the two described competing effects. Identifying an optimal lipophilicity window could ensure sufficient partitioning to establish a stable electrode potential without introducing excessive amounts of the organic salt into the sample or confining its ions to the organic phase at the risk of inducing parasitic responses. Nevertheless, this opportunity remains relatively underexplored. Most recent studies continue to focus on moderately lipophilic ILs,^10,11,13,25–29^ while more lipophilic salts, when considered, are quickly dismissed without further assessment owing to their relatively poorer performance.^30^

Herein we address this gap by critically evaluating the performance of REs based on ILs with increased lipophilicity compared to commonly used commercial analogs. Three different ILs were synthesized in-house via potentiometric titrations of commercially available cations and anions and subsequently incorporated into conventional polymeric reference membranes based on poly­(vinyl chloride) (PVC) and dodecyl 2-nitrophenyl ether (NPDE). The best-performing composition exhibited stable electrode potentials largely independent of sample composition, combined with significantly reduced electrolyte leaching and complete elimination of ISE membrane poisoning in small sample volumes. These findings emphasize the need for a systematic reevaluation of the lipophilicity of ILs and organic electrolytes used in the design of REs.

## Experimental Section

### Chemicals

The ion-selective and reference membrane components poly­(vinyl chloride) (PVC, Selectophore), 2-nitrophenyl dodecyl ether (NPDE, Selectophore), bis­(2-ethylhexyl)­sebacate (DOS, 97%), sodium tetrakis­(3,5-bis­(trifluoromethyl)­phenyl)­borate (NaTFPB, Selectophore), valinomycin (Selectophore) and tetrahydrofuran (THF, Selectophore) were purchased from Sigma-Aldrich. Octadecyl amine-functionalized single-walled carbon nanotubes (f-SWCNTs, 80–90% carbon basis, *D* × *L*: 2–10 nm × 0.5–2 μm) were also purchased from Sigma-Aldrich. Precursors for ionic liquid preparation tetrahexylammonium bromide (THABr, 99%), tetraheptylammonium bromide (THeptABr, 99%), tetraoctylammonium bromide (TOABr, 98%) and sodium bis­(trifluoromethanesulfonyl)­imide (Na­[C_1_C_1_N^–^], 97%) were sourced from Sigma-Aldrich. Solvents for ionic liquid synthesis via potentiometric titrations 1,2-dichloroethane (DCE, 99.8%), ethyl acetate (EtOAc, HPLC grade) and dichloromethane (DCM, HPLC grade) were obtained from Sigma-Aldrich (DCE) and Fisher Chemical (EtOAc, DCM). Ionic liquid tributyl­(2-methoxyethyl)­phosphonium bis­(trifluoromethanesulfonyl)­imide ([TBMOEP^+^]­[C_1_C_1_N^–^]) was purchased from abcr. pH of blood electrolyte solutions was adjusted using volumetric 1 M solutions of hydrochloric acid (HCl) and sodium hydroxide (NaOH) acquired from Thermo Scientific. Sodium chloride (NaCl), sodium sulfate (Na_2_SO_4_), sodium acetate (NaOAc), sodium nitrate (NaNO_3_), sodium phosphate monobasic (NaH_2_PO_4_), sodium phosphate dibasic (Na_2_HPO_4_), potassium chloride (KCl), calcium chloride (CaCl_2_) and magnesium chloride (MgCl_2_) were all purchased from Sigma-Aldrich. All salts used were analytical grade or higher. All aqueous solutions were prepared with deionized water (>18.2 MΩ·cm).

### Instrumentation

All potentiometric measurements were performed with a high impedance 16 channel EMF monitor (Lawson Laboratories, Malvern, PA) using a double-junction Ag/AgCl/3 M KCl/1 M LiOAc reference electrode (Metrohm, Switzerland) unless otherwise stated. All emf values were corrected for the liquid junction potential of the commercial RE.^31^ pH of blood electrolyte solutions was adjusted with 827 pH lab (Metrohm, Switzerland). Temperature calibrations were performed with a Dyneo DD-450F thermostat (Julabo, Germany) connected to an external thermostated cell. Quartz crystal microbalance (QCM, QSense Analyzer, Biolin Scientific, Sweden) was used to compare the rates of ionic liquid leaching from reference membranes.

### Ionic Liquid Preparation via Potentiometric Titrations

Ionic liquids with precisely matched concentrations of lipophilic cations and anions were synthesized following a previously published synthetic procedure^24^ with slight modifications. The experimental setup used in this study is shown in Figure 1a. Titrations were carried out in a stirred solution at room temperature under a fume hood. In-house prepared Ag/AgBr wires were used as “working” and “reference” electrodes. Both electrodes were inserted into glass bodies sealed on the opposite end with microporous polypropylene membranes Celgard 2500 (Celgard, Charlotte, NC) which were held in place by rubber o-rings. The electrode bodies were filled with aqueous internal solutions: 1 M NaBr for the “reference” electrode and 10 mM NaBr for the “working” electrode. Sample solutions (20 mL) contained 2.5 mM THABr/THeptABr/TOABr dissolved in DCE/EtOAc 1:1 additionally topped with 5 mL of deionized water. 8 mM Na­[C_1_C_1_N^–^] dissolved in the same solvent mixture was used as the titrant. Titrations were performed by adding the titrant to the sample using a glass syringe mounted onto a syringe pump operating at 0.5 mL min^–1^. The electrodes and the stainless steel needle of the syringe were immersed directly into the organic layer.

**Figure 1:**
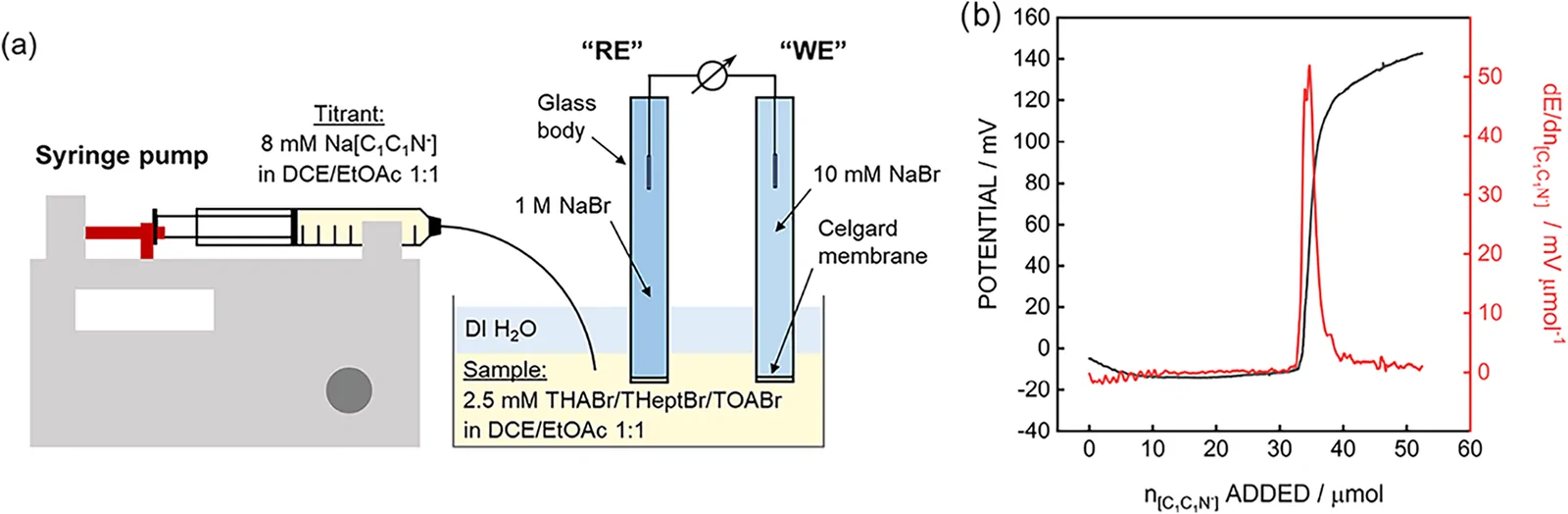
(a) Experimental setup used for the potentiometric titration experiments and (b) an experimental potentiometric titration curve obtained during the synthesis of [TOA^+^]­[C_1_C_1_N^–^].

Initially, three replicate full titrations of the corresponding organic cation solution with the organic anion solution were performed to accurately determine the titration end point expressed as the time from the experiment start and, therefore, the corresponding titrant volume. The end point was identified through differentiation of the potentiometric titration curve (Figure 1b). Using the same standardized sample and titrant solutions, preparative titrations were then carried out, which were stopped at the volume corresponding to the previously found end point. These were repeated 3–5 times, after which the titrated mixtures were combined and transferred into a separatory flask. The organic and aqueous fractions were separated. The aqueous layer was washed with DCM (3 × 25 mL). The joint organic fraction was washed with 50 mL brine and dried over a minimal possible amount of MgSO_4_. The drying agent was then filtered off and the solvent rotary evaporated. All electrolytes were obtained as transparent viscous liquids with the following yields [THA^+^]­[C_1_C_1_N^–^] – 115 mg (91%, 4 preparative titrations), [THeptA^+^]­[C_1_C_1_N^–^] – 107 mg (quantitative, 3 titrations), [TOA^+^]­[C_1_C_1_N^–^] – 162 mg (86%, 5 titrations).

### Electrode Preparation and Calibration

Reference and ion-selective membrane compositions were prepared by weighing sensing components together with PVC and plasticizer to obtain a total cocktail mass of 250 mg, which was then dissolved in 2 mL THF. The membrane compositions used throughout the study are given in Table 1

**Table 1:** Reference and Ion-Selective Membrane Compositions Used Throughout This Study^d^

	Membrane components								
Membrane	PVC^a^	NPDE^a^	DOS^a^	[THA^+^][C_1_C_1_N^–^]^b^	[THeptA^+^][C_1_C_1_N^–^]^b^	[TOA^+^][C_1_C_1_N^–^]^b^	[TBMOEP^+^][C_1_C_1_N^–^]^b^	NaTFPB^b^	Valinomycin^b^
M1	48.4	48.4	-	50	-	-	-	-	-
M2	48.3	48.3	-	-	50	-	-	-	-
M3	48.1	48.1	-	-	-	50	-	-	-
M4	48.7	48.7	-	-	-	-	50	-	-
M5^c^	32.6	-	65.3	-	-	-	-	5	15

a Mass percentage.

b mmol kg^–1^.

c Total cocktail mass of 200 mg in 2 mL THF.

d The amounts are relative to a total cocktail mass of 250 mg dissolved in 2 mL THF.

Reference membranes for electrodes with an internal filling were cast by pouring the corresponding membrane compositions (M1–3) into a 2.2 cm-diameter glass ring affixed on a glass slide. The solvent was allowed to evaporate overnight. Three 8 mm disks were cut out of the resulting parent membrane, mounted in commercial electrode bodies (Oesch Sensor Technology, Sargans, Switzerland), filled with 1 mM KCl and conditioned in the same solution overnight.

Solid-contact reference electrodes (REs) and K^+^-selective ion-selective electrodes (ISEs) were prepared using commercial glassy carbon macroelectrodes (electrode diameter 3 mm, total diameter including casing 1 cm) and glassy carbon electrodes made in-house (electrode diameter and total diameter 3 mm), respectively. Prior to deposition of the transducing layer and the membrane, the electrodes were polished with diamond sprays (Kemet International, UK) of 6, 3, 1, and 0.25 μm particle size. f-SWCNTs were employed as the transducing material, the suspension for drop-casting was prepared by dispersing 1 mg of f-SWCNTs in 1 mL THF.

Solid-contact REs were prepared by drop-casting 5 successive layers (20 μL each) of the f-SWCNTs suspension, allowing 10 min of drying between each deposition. The completed transducing layer was dried for 20 min prior to membrane deposition. Reference membranes were then formed by drop-casting 3 layers (50 μL each) of the membrane composition M1 with 20 min drying following the deposition of each layer. After the drop-casting was complete, the electrodes were first left to dry overnight followed by overnight conditioning in 1 mM KCl.

Solid-contact K^+^-ISEs were prepared following the same general procedure for solid-contact REs with reduced volumes of the transducer suspension (2 μL) and the membrane composition M5 (5 μL) to accommodate the reduced total electrode surface area. ISEs were conditioned in 1 mM KCl prior to use.

During all electrode calibrations, the conditioned electrodes were first immersed into the most dilute solution followed by stepwise addition of aliquots of the analyte standard solution to the analyzed solution at 2 min intervals. Each calibration was repeated 3 times using the same electrodes.

### Temperature Calibrations of Reference Electrodes

Temperature calibrations were performed in a thermostated cell filled with 230 mL of 1 mM KCl unless otherwise stated, using a commercial Ag/AgCl wire (Metrohm, Switzerland) as reference electrode. All REs were prepared according to the general procedure described above. Both REs with an internal filling and solid-contact REs were calibrated simultaneously against the same reference by applying a temperature cycle, which was repeated 3 times.

### Flow Experiments

Flow experiments were carried out using a wall-jet cell design and overall flow system configuration described previously.^32^ Three electrodes were inserted into the cells and placed in a 250 mL beaker at equal distances from a commercial Ag/AgCl/3 M KCl/1 M LiOAc reference electrode. The beaker was filled with 170 mL of 3 M KCl. Sample solutions were delivered to the system, and the bulk electrolyte was continuously renewed using peristaltic pumps equipped with ISMATEC tygon tubings (inner diameter 1.42 mm, wall 0.86 mm), which were connected to a flow path composed of PTFE tubings (inner diameter 0.75 mm, BOLA) and bubble traps (for sample delivery only). Pump 1 supplied the sample solutions to the cells at a flow rate of 0.4 mL min^–1^ per channel (total flow rate 1.2 mL min^–1^). The dead time, *i.e*. time required for the sample to reach the electrode cells, was approximately 2.5 min. Pump 2 delivered 3 M KCl to the bottom of the beaker at a flow rate of 1.2 mL min^–1^, while Pump 3, set to 2.4 mL min^–1^ (corresponding to the combined inflow from Pumps 1 and 2), was used to remove excess electrolyte from the top of the beaker.

### QCM Study

The leaching rates of ionic liquids from the membranes were compared using quartz crystal microbalance. Membrane compositions M1 and M4 were diluted in THF (dilution factor of 4). Afterward, 25 μL of the diluted membrane compositions were spin-coated onto silicon dioxide QCM sensors (active area 0.38 cm^2^, QSX303, Biolin Scientific, Sweden) at 1500 rpm for 2 min to obtain thin reference membranes. The modified QCM sensors were mounted into the stock measurement cell of the instrument, all measurements were performed at 25 °C. The seventh overtone frequency was selected as the analytical signal. After approximately 20 min of temperature equilibration under dry conditions, a 1 mM KCl solution was introduced into the cell at a flow rate of 0.5 mL min^–1^, and frequency changes were then monitored for 1.5 h.

### Ion-Selective Membrane Poisoning Experiments

Ion-selective membrane poisoning tests were conducted in a small-volume cell containing 1.5 mL of 10 mM KCl under continuous stirring. The potential of a K^+^-ISE was measured against a commercial Ag/AgCl wire reference electrode (Metrohm, Switzerland). After an initial stabilization for 6–7 min, an unconditioned 8 mm reference membrane disk (compositions M1 or M4) was inserted into the sample perpendicular to the ISE surface, and the emf was recorded for an additional 20 min. Different K^+^-ISEs from the same batch were used for the experiments with each of the reference membranes.

## Results and Discussion

### Ionic Liquid Structure Screening

The ILs were synthesized using a modified version of a previously reported preparative potentiometric titration method,^24^ which allows one to precisely match the concentrations of the constituent ions in the final product. Tetraalkylammonium cations and bis­(perfluoroalkanesulfonyl)­imide anions were selected to ensure that the general physicochemical properties of the synthesized ILs remain comparable to those of commonly used commercial analogues. Owing to the limited availability of derivatives with extended side chains, bis­(trifluoromethanesulfonyl)­imide ([C_1_C_1_N^–^]) found across most typically used ILs was employed as the common anion in this study as well (Figure 2). The lipophilicity of the ILs was tuned by using tetraalkylammonium cations with varying length of the aliphatic chains: tetrahexylammonium ([THA^+^]), tetraheptylammonium ([THeptA^+^]) and tetraoctylammonium ([TOA^+^]) (Figure 2). Although the electrochemical properties of [THA^+^]­[C_1_C_1_N^–^] have been investigated by Kakiuchi and co-workers,^33–36^ the ionic liquids proposed here have not yet been applied in RE design.

**Figure 2:**
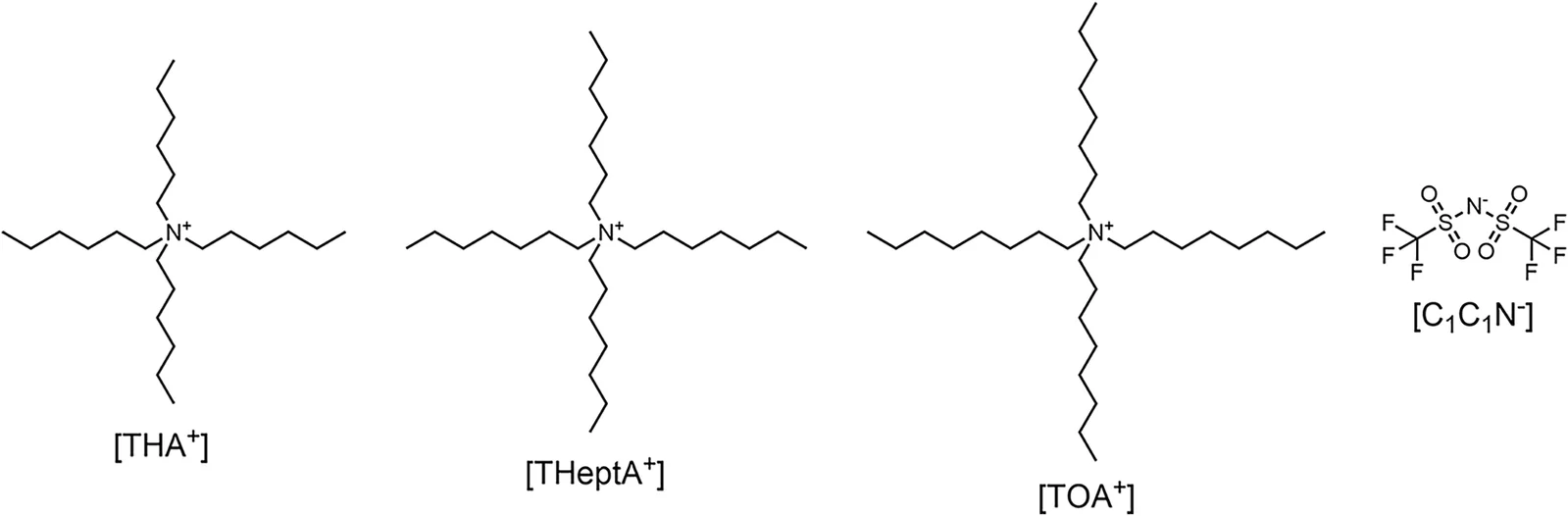
Structures of the ions composing lipophilic salts used in this study.

Although the logarithmic partition constants are challenging to determine experimentally with sufficient precision, they could be estimated for the PVC/NPDE 1:1 reference membrane matrix employed in this work. The corresponding values are 8.6 for [THA^+^]­[C_1_C_1_N^–^], 10.6 for [THeptA^+^]­[C_1_C_1_N^–^] and 12.6 for [TOA^+^]­[C_1_C_1_N^–^] (see Supporting Information for more detail). These values exceed those of commonly used ILs such as 1-methyl-3-octylimidazolium bis­(trifluoromethanesulfonyl)­imide ([C_8_mim^+^]­[C_1_C_1_N^–^], 3.6) and tributyl­(2-methoxyethyl)­phosphonium bis­(trifluoromethanesulfonyl)­imide ([TBMOEP^+^]­[C_1_C_1_N^–^], 4.7) by several orders of magnitude. Conveniently, this lipophilicity increase translates to a substantially reduced equilibrium concentration of the IL in the aqueous phase. For a membrane doped with 50 mmol kg^–1^ of [C_8_mim^+^]­[C_1_C_1_N^–^], the expected concentration of the IL in the vicinity of the membrane is approximately 0.8 mM. In contrast, for [THA^+^]­[C_1_C_1_N^–^] this value already decreases by a factor of 320 to about 2.5 μM and is further reduced to approximately 0.25 and 0.025 μM for [THeptA^+^]­[C_1_C_1_N^–^] and [TOA^+^]­[C_1_C_1_N^–^], respectively. While such reduced partitioning is clearly advantageous in terms of minimizing sample contamination, it must remain sufficiently high to ensure stability and sample-independence of electrode potential.

The performance of conventional liquid-contact REs based on the synthesized ILs was evaluated in KCl solutions ranging from 10 μM to 100 mM (Figure 3). The results strongly depended on the lipophilicity of the used electrolyte. Electrodes incorporating the least lipophilic [THA^+^]­[C_1_C_1_N^–^] exhibited excellent potential stability across the entire concentration range, with a standard deviation of the RE potential of ±1.2 mV across 27 measurements, for a total potential spread of 3.8 mV. In contrast, increasing electrolyte lipophilicity led to deterioration of the RE function. Reference membranes doped with [THeptA^+^]­[C_1_C_1_N^–^] demonstrated a standard deviation of ±2.8 mV and a potential spread of 9.5 mV across three consecutive calibrations, with particularly pronounced instability in highly concentrated solutions. The most lipophilic [TOA^+^]­[C_1_C_1_N^–^] yielded the poorest performance: the RE potential in dilute samples was highly irreproducible (±14.4 mV standard deviation and 37.3 mV of total variation), whereas further increasing the KCl concentration induced a measurable response to the sample composition. As recently discussed by Dong et al.,^29^ this behavior likely originates from the coextraction of the IL cation back into the membrane together with the sample anion, driven by the inherent imbalance of constituent ion lipophilicities in the ILs used in this study. The RE potential occasionally exhibited small spikes upon addition of analyte stock solution aliquots similar to observations from our previous studies.^24^ Interestingly, this behavior, likely of kinetic origin, did not change significantly with increasing reference electrolyte lipophilicity, and the response time of the REs generally remained at or below 1 min. Overall, the RE stability data reveal a clear trend: increasing the lipophilicity of the potential-determining electrolyte negatively affects the electrode potential stability and reproducibility, which may be attributed to the increasing influence of the sample species on the interfacial equilibria as the IL fraction in the sample decreases.

**Figure 3:**
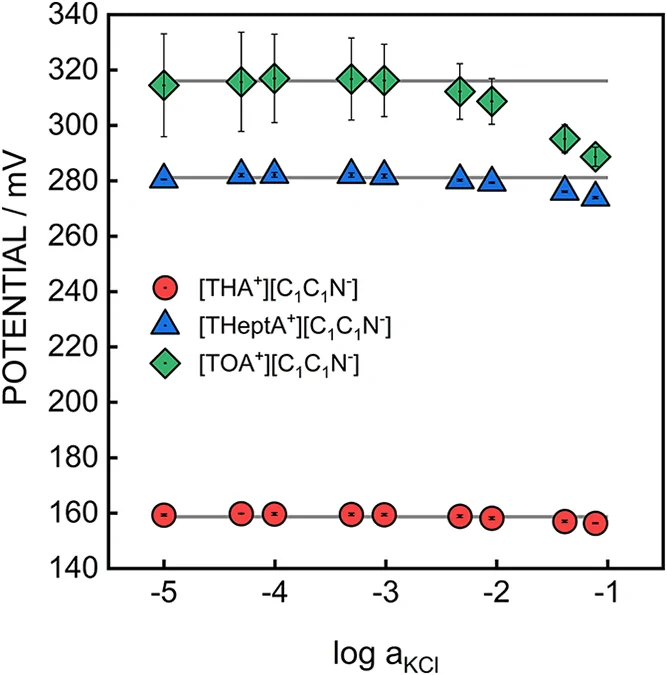
Response of REs based on [THA^+^]­[C_1_C_1_N^–^] (membrane M1), [THeptA^+^]­[C_1_C_1_N^–^] (membrane M2) and [TOA^+^]­[C_1_C_1_N^–^] (membrane M3) in KCl solutions. Shape center dots show the measured emf and error bars are standard deviations (*n* = 3) acquired across three consecutive calibration replicates with the same electrode. The solid lines represent a stable potential plateau for each electrode type.

The RE standard potentials exhibited an upward shift with increasing lipophilicity of the reference electrolyte (Figure 3), which is inconsistent with the symmetrical nature of the liquid-contact RE design. Analogous partitioning processes at the membrane interfaces facing the sample and the internal filling solution should generate interfacial potentials of identical magnitude and opposite sign, which compensate each other to yield an overall membrane potential near 0 mV. The observed deviation from this expected behavior may originate from the membrane conditioning protocol employed in this work. The reference membranes were conditioned directly in the electrode bodies, after which the outer conditioning solution was replaced with sample solutions of interest, whereas the inner filling solution remained unchanged throughout both the conditioning and all subsequent measurements. As a result, impurities removed from the membrane during the conditioning step, most notably any excess of one of the reference electrolyte constituent ions, remained confined to the inner filling solution and may therefore substantially influence the respective interfacial potential. To test this hypothesis, calibrations of REs based on [THA^+^]­[C_1_C_1_N^–^] were performed immediately after conditioning, which were followed by multiple cycles of flipping the reference membrane, renewing the inner filling solution and recalibrating the electrode. The corresponding calibration curves shown in Figure S1 exhibited a potential shift upon replacement of the original inner filling solution, while the RE behavior remained largely unchanged during the subsequent cycles. These results confirm the influence of the composition of the solution at the backside of the membrane on the overall electrode potential. Nevertheless, other mechanisms may contribute to the observed potential shifts, including concentration polarization within the membrane or at the membrane interfaces with the sample and the internal filling solution. Distinguishing between these contributions requires further investigation and falls outside of the scope of the present practically oriented study.

### Response of Reference Membranes Doped with [THA^+^]­[C_1_C_1_N^–^] to Aqueous Electrolytes

Since [THA^+^]­[C_1_C_1_N^–^] emerged as the ionic liquid most suitable for RE design out of the three tested compositions, the corresponding REs were subjected to further evaluation in the presence of various aqueous electrolytes (Figure 4). The corresponding response characteristics are summarized in Table 2. The RE potential remained consistently stable in a wide range of electrolyte solutions. The observed response slopes were generally limited to within ±1 mV, with slightly higher values of −2.1 mV for NaOAc and −1.6 mV for NaNO_3_. The average potential of the REs was also highly reproducible across different aqueous electrolytes, with the maximum difference of 3.1 mV observed between NaCl and NaNO_3_. Varying the ionic strength of the sample induced minor changes in the electrode potential over the course of the calibration. The potential standard deviation observed within the investigated concentration range of 10 μM–100 mM mostly remained at or below ± 1.8 mV, with NaOAc presenting a slight deviation at ± 2.5 mV. Overall, the general performance of the REs based on [THA^+^]­[C_1_C_1_N^–^] was consistent with the previous reports on moderately lipophilic ionic liquid REs.^13,22,25,30,37,38^ However, a notable departure from this trend was observed in highly concentrated NaNO_3_ solutions. At concentrations of 10 mM or higher, the RE potential began to deviate significantly from the plateau value and a response slope of −15.4 ± 0.4 mV toward nitrate ions emerged. Similar to the chloride response previously observed with [TOA^+^]­[C_1_C_1_N^–^]-based REs, this behavior likely originates from the favorable coextraction of the IL cation into the membrane with the more lipophilic sample anion, facilitated by the substantially higher lipophilicity of the IL cation relative to its counterion. Although not likely to pose limitations for analyses in the presence of naturally occurring nitrate backgrounds,^39^ it highlights an important drawback of the IL compositions used in this study. While the electrode performance is satisfactory in solutions of most inorganic electrolytes, the balance of IL constituent ion lipophilicity needs to be further optimized to extend the operational range of the corresponding REs to samples containing more lipophilic anionic species.

**Figure 4:**
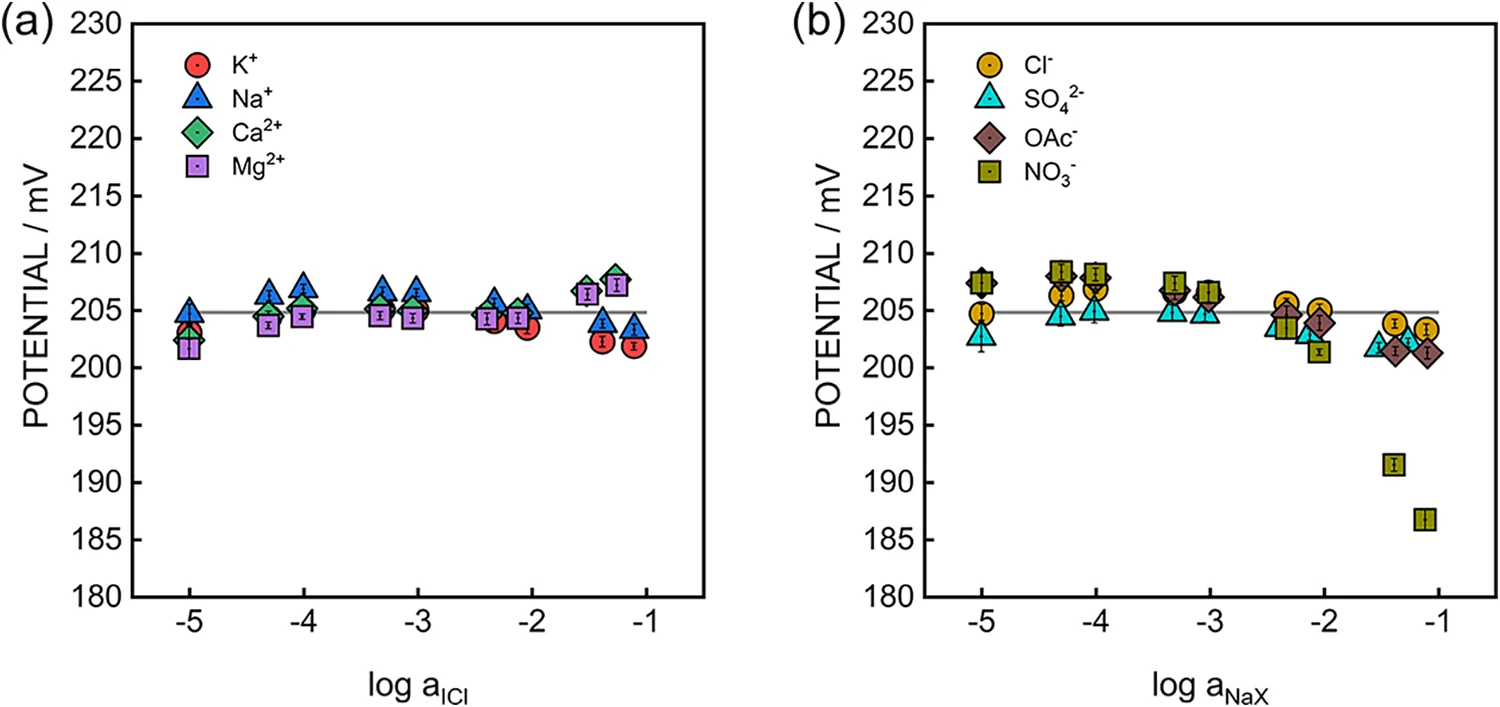
Response of an RE based on [THA^+^]­[C_1_C_1_N^–^] (membrane M1) in solutions of (a) chloride salts of various cations (I^+^) and (b) sodium salts of various anions (X^–^). Shape center dots show the measured emf and error bars are standard deviations (*n* = 3) acquired across three consecutive calibration replicates with the same electrode. The solid lines represent the potential plateau average value across all tested electrolyte solutions.

**Table 2:** Characteristics of [THA^+^]­[C_1_C_1_N^–^]-Based Liquid-Contact RE Response in Pure Electrolyte Solutions from 10 μM to 100 mM

	Electrolyte						
Parameter	KCl	NaCl	CaCl_2_	MgCl_2_	Na_2_SO_4_	NaOAc	NaNO_3_ ^a^
Slope, mV	–0.7 ± 0.3	–0.9 ± 0.3	0.4 ± 0.3	0.8 ± 0.2	–0.9 ± 0.2	–2.1 ± 0.2	–1.6 ± 0.6
Average potential, mV	203.8	205.4	205.1	204.6	203.6	205.3	206.9
Standard deviation, mV	±1.2	±1.3	±1.4	±1.5	±1.3	±2.5	±1.8
Maximum potential variation^b^, mV	4.3	4.4	7.0	7.1	4.6	8.1	6.3

a From 10 μM to 5 mM NaNO_3_.

b Difference between the maximum and minimum potential values obtained across 27 measurements.

Following the initial tests with liquid-contact REs, the proposed reference membranes were integrated into a solid-contact configuration as well. Using functionalized single-walled carbon nanotubes as the transducing material, solid-contact REs based on [THA^+^]­[C_1_C_1_N^–^] were obtained, which exhibited performance on par with their analogues with an internal filling solution. The RE slope in NaCl solutions from 10 μM to 1 M was only −1.3 mV, while the standard deviation across 5 orders of magnitude of NaCl concentration was ±1.9 mV with a maximum potential variation of 6.9 mV (Figure S2).

### Temperature Dependence of Reference Electrodes

The response of both the liquid-contact and the solid-contact REs was subsequently studied as a function of temperature. This performance aspect of REs based on organic electrolytes is often overlooked, however, their application beyond strictly controlled laboratory environment requires a clear understanding of the effect of temperature on their operation. For the sake of simplicity, temperature calibrations were performed using a commercial Ag/AgCl wire as a reference electrode. During the measurements, the liquid-contact and solid-contact REs, together with the common Ag/AgCl wire reference, were immersed into 1 mM KCl contained in a closed thermostated cell. As before, the liquid contact REs were also filled with 1 mM KCl, thus forming a symmetrical system in which the reference membrane was placed between two Ag/AgCl elements immersed in identical KCl solutions. The temperature was varied from 25 to 5 °C in 5 °C increments, the cycle was repeated three times. Where necessary, the temperature-dependent contribution of the Ag/AgCl reference was corrected by assuming a Nernstian response. The resulting liquid-contact and solid-contact RE temperature calibrations for one representative measurement cycle are presented in Figure 5, while the corresponding raw potential–time traces for the complete electrochemical cells are shown in Figure S3.

**Figure 5:**
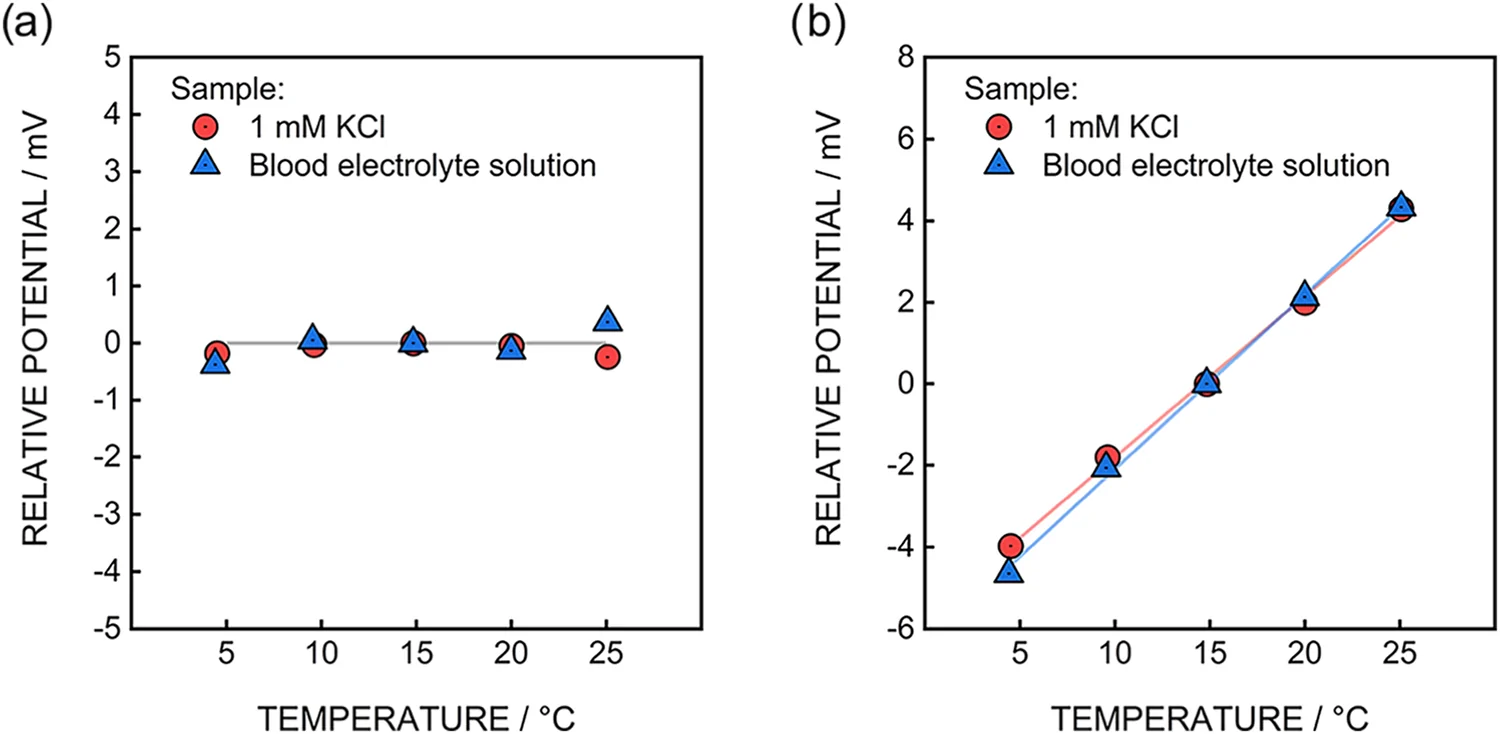
Response of (a) liquid-contact and (b) solid-contact REs based on [THA^+^]­[C_1_C_1_N^–^] (membrane M1) in 1 mM KCl and a blood electrolyte solution (see Table 3, solution “HIGH”) against an Ag/AgCl wire reference electrode at different temperatures. Shape center dots show the measured emf. The values correspond to the third and final cycle of the respective temperature ramp experiments (Figures S3–S4), the temperature-dependent contribution of the Ag/AgCl wire reference was corrected based on the sample solution composition assuming Nernstian response.

Since a reference membrane in a liquid-contact RE constitutes a symmetrical system with two largely identical interfaces, temperature effects at the individual interfaces are expected to cancel, resulting in minimal temperature dependence of the overall membrane potential. Indeed, such behavior was observed experimentally with the total potential variation across a single temperature cycle not exceeding 0.9 mV (Figure 5a, red circles). Temperature transitions from 5 to 25 °C between successive cycles resulted in more pronounced potential drops of approximately 1.6 mV, however, the slight upward drift suggests that this effect may originate from the inefficient heat transfer between the external sample solution and the interior of the electrode bodies (Figure S3a). In contrast, solid contact REs, in which the membrane is inherently asymmetric due to facing the sample on one side and the transducer on the other, exhibited a stronger dependence of the potential on temperature, with a standard deviation of ±2.9 mV (overall change up to 8.3 mV), corresponding to a response slope of 0.39 mV K^–1^ (Figure 5b, red circles). Although the positive potential-temperature slope is consistent with expectations for REs based on organic electrolytes (see ref 16 for further details), the limited understanding of the effect of temperature changes on the transducing material complicates further theoretical interpretation of the observed behavior. The temperature dependence study was subsequently expanded by substituting the 1 mM KCl sample with a blood electrolyte solution with potassium, sodium, chloride and phosphate concentrations and pH at the upper limits of their respective clinical ranges (see Table 3, “HIGH”). The RE behavior remained largely unaffected by this change (Figures 5 and S4). The liquid contact REs, now filled with 0.1 M KCl, retained a stable and essentially temperature independent potential close to 0 mV (Figure 5a, blue triangles), whereas solid-contact REs exhibited only a negligible variation of the potential–temperature slope from 0.39 mV K^–1^ in 1 mM KCl to 0.43 mV K^–1^ in the blood electrolyte solution. These results demonstrate the robustness of the proposed electrodes under variable ambient temperature conditions.

**Table 3:** Compositions of Artificial Blood Electrolytes Used in This Study, Based on Ref 13

	Electrolytes				
Blood electrolyte solution	KCl	NaCl	Na_2_HPO_4_	NaH_2_PO_4_	pH
LOW	3.5 mM	96.4 mM	0.74 mM	0.18 mM	7.35
HIGH	4.7 mM	102.8 mM	0.82 mM	0.26 mM	7.45

Similar temperature calibrations were performed using a commercial double-junction reference electrode instead of an Ag/AgCl wire. In this experiment, 1 and 3 M KCl were used as reference and bridge electrolytes, respectively, while liquid-contact REs were filled with 1 M NaCl to match the environment of the Ag/AgCl elements and thereby compensate for their contribution to the temperature-dependent changes in cell emf. Nevertheless, more pronounced potential variations were observed with this setup for both liquid-contact and solid-contact REs (Figure S5), with standard deviations across a single temperature cycle of approximately ±4.7 mV (potential spread of 13.4 mV; slope of 0.62 mV K^–1^) for the former and ±3.9 mV (potential spread of 11.3 mV; slope of 0.47 mV K^–1^) for the latter. This is most likely a consequence of particularly poor heat transfer from the outside sample solution to the inner compartment of the reference electrode housing the Ag/AgCl element. To further illustrate the importance of efficient temperature equilibration in temperature dependence studies, an additional experiment was performed using the symmetrical setup shown in Figure S6a. Two Ag/AgCl wires were placed inside a sealed U-shaped glass tube filled with 1 mM KCl. One wire (the "working" electrode) was immersed into a thermostated Milli-Q water bath cell together with the lower part of the tube, whereas the other wire (the "reference" electrode) stayed approximately 7 cm above the bath level. An identical temperature ramp was applied, and the resulting potential-time trace is shown in Figure S6b. Despite both Ag/AgCl wires being immersed in the same solution within the same glass tube, the temperature difference between the two corresponding areas of the vessel were sufficient to generate a potential difference of approximately 13 mV between 25 and 5 °C in an otherwise fully symmetrical electrochemical cell. These results indicate that incomplete temperature equilibration alone may produce significant potential variations similar to those observed with the commercial double-junction RE. The results of temperature studies performed using the latter should therefore be interpreted with caution, as they likely reflect not only the intrinsic electrochemical behavior but also thermal gradients within the measurement setup.

### Reference Electrode Stability under Flow Conditions

Solid-contact REs based on [THA^+^]­[C_1_C_1_N^–^] were additionally tested under flow conditions. This approach allowed one to detect potential transients induced by abrupt changes in the aqueous environment. The electrodes were tested using a wall-jet cell design (see Experimental Section for details) at a constant flow rate of 0.4 mL min^–1^. The RE response to a sequence of various electrolyte solutions (5 mM KCl, 10 mM MgCl_2_, 1 mM Na_2_SO_4_, 100 mM NaCl, 1 mM NaOAc and 5 mM CaCl_2_) under flow conditions is shown in Figure 6a. The standard deviation of the RE potential across the tested solutions was ±1.6 mV (total variation 4.5 mV), which agreed well with the behavior observed in bulk solutions under constant stirring (Figure 4 and Table 2). Notably, transient potential spikes were observed upon transitions between certain electrolytes. This behavior resembles a phenomenon previously reported for ionophore-based REs and likely arises from the reference electrolyte component diffusion within the membrane and in its vicinity.^22,24,40,41^ In the present case, the nature of the aqueous electrolyte appears to be the primary driving factor rather than its concentration. Elucidating the precise origin of this effect will require further investigation and falls outside the scope of this work as it does not significantly affect the overall usability of the proposed REs.

**Figure 6:**
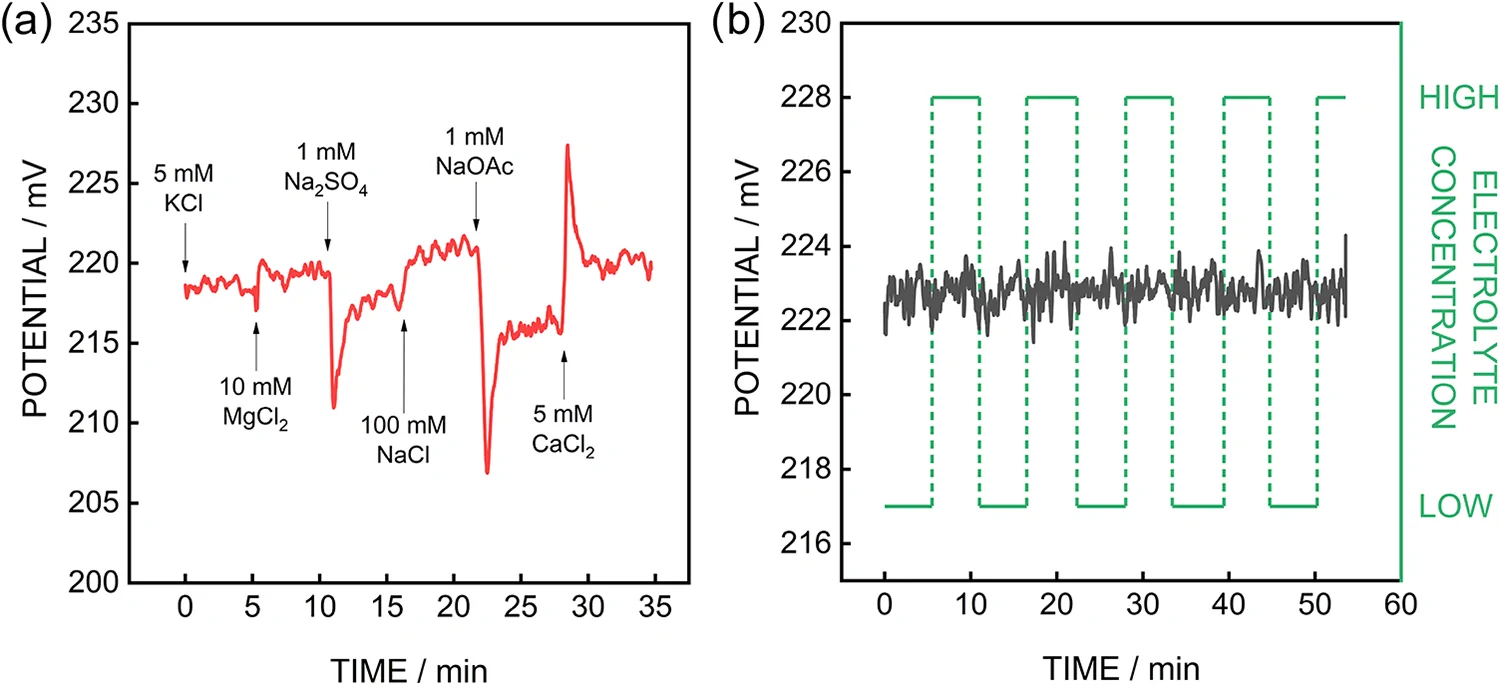
Potential-time traces for a solid-contact RE based on [THA^+^]­[C_1_C_1_N^–^] (membrane M1) under flow conditions in the presence of (a) a sequence of various aqueous electrolytes at different concentrations and (b) artificial blood electrolyte solutions of high and low concentrations (see Table 3 for exact compositions). High-frequency noise arising from the flow conditions was filtered by adjacent averaging using a 10-point window.

Exposure of the REs to samples spanning diverse electrolytes and concentration levels provides valuable insight, but it does not accurately reflect realistic application conditions. To simulate them, we employed the flow system to assess the RE potential stability in artificial blood electrolyte solutions. Key parameters, such as pH and the levels of potassium, sodium, chloride and phosphate, were varied from the lower (solution designated as LOW) to the upper (solution designated as HIGH) limits of their respective clinical ranges (Table 3). This experiment was inspired by the work of Chen et al.^13^ As evident from the corresponding potential trace (Figure 6b), alternating between the two blood electrolyte solutions did not produce any appreciable change in electrode potential. The average potential variation between the two samples was merely 160 μV, which corresponds to a measurement error of 0.62% for monovalent ions and 1.25% for divalent ions. These results suggest attractive RE performance in a more realistic practical scenario.

### Evaluation of Electrolyte Leaching and ISE Membrane Poisoning

While achieving stable RE potentials was an important step, the primary goal of this study is to combine such stability with reduced leaching of the reference electrolyte into the sample. Quartz crystal microbalance (QCM) measurements were employed to assess the latter, as shown in Figure 7. Thin reference membranes containing [THA^+^]­[C_1_C_1_N^–^] were spin-coated onto the resonator, and film mass changes, expressed as shifts in resonator frequency, were monitored upon its exposure to 1 mM KCl. After the solution flow start and the initial equilibration, the resonator frequency remained highly stable throughout the rest of the measurement (Figure 7, red trace), indicating minimal film mass loss due to leaching. For comparison, films based on the same matrix but doped with the commercial [TBMOEP^+^]­[C_1_C_1_N^–^] were also evaluated. In this case, the corresponding frequency trace began rising after stabilizing (Figure 7, blue trace), indicating a substantial reduction of the film mass due to electrolyte loss. While these results are encouraging, it is important to highlight the limitations of quantitative QCM estimation of the electrolyte leaching from such membranes. The overall stabilization of the quartz resonator and gradual water uptake into the membrane upon transition from air to 1 mM KCl severely complicate accurate monitoring of the initial distribution of the organic salt between phases, which is established immediately upon first contact with solution. Given the extremely small film thickness (ca. 200 nm^42^), a large fraction of the electrolyte is expected to be lost almost immediately. Consequently, the observed leaching rates are not representative of thicker reference membranes used in macroelectrodes. In addition to that, the studied films exhibited pronounced viscoelastic character reflected in the frequency response variation between different overtones (data not shown). Hence, the Sauerbrey equation cannot be directly applied to transform the observed frequency response changes into film mass information. Together, all these constraints restricted the QCM use in this study to qualitative assessment only.

**Figure 7:**
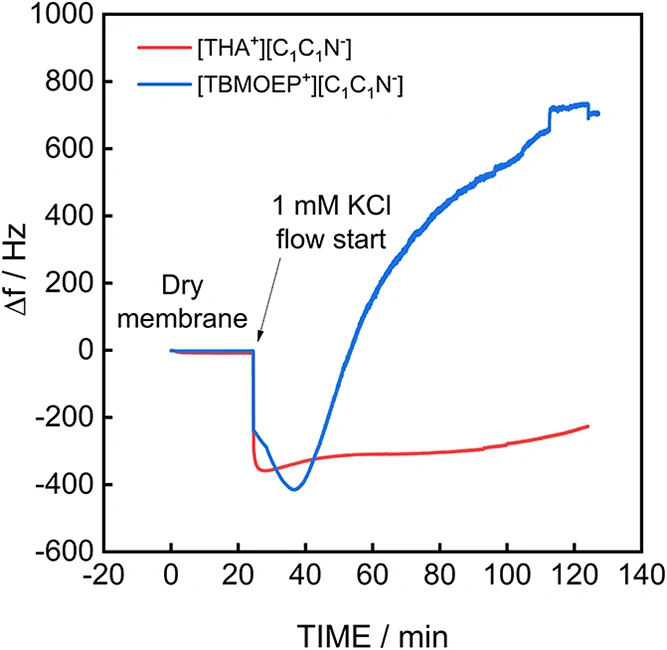
Frequency changes observed by QCM upon exposure of thin reference membranes based on [THA^+^]­[C_1_C_1_N^–^] (membrane M1) and [TBMOEP^+^]­[C_1_C_1_N^–^] (membrane M4) to 1 mM KCl.

The reduced electrolyte leaching observed for [THA^+^]­[C_1_C_1_N^–^] limits sample contamination and thereby should mitigate the issue of possible ISE membrane poisoning. In turn, this broadens the scope of feasible applications toward analyses involving small sample volumes. To demonstrate this advantage, we performed K^+^-ISE poisoning experiments in the presence of reference membranes based on [THA^+^]­[C_1_C_1_N^–^] and [TBMOEP^+^]­[C_1_C_1_N^–^] (Figure 8). A potassium-selective electrode was immersed into 1.5 mL of 10 mM KCl together with an Ag/AgCl wire serving as a reference. The emf was recorded before and after inserting a reference membrane disk into the solution. The surface area and the thickness of the membrane disk corresponded to those of typical reference membranes used in liquid-contact and solid-contact REs throughout this study. As expected, introducing the membrane containing [THA^+^]­[C_1_C_1_N^–^] into the sample did not affect the potential of the ISE. On the contrary, adding a membrane doped with [TBMOEP^+^]­[C_1_C_1_N^–^] induced an instantaneous potential drift, amounting to nearly 100 mV over the following 20 min. This experimental evidence confirms that, unlike with commonly used commercial ILs, the equilibrium aqueous concentration of [THA^+^]­[C_1_C_1_N^–^] is sufficiently low to prevent any undesired interference with polymeric ISEs.

**Figure 8:**
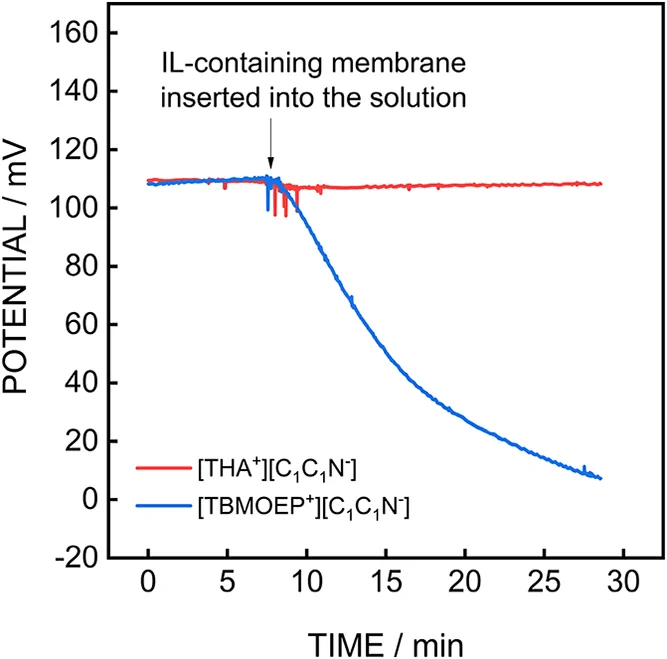
Potential traces of solid-contact K^+^-ISEs (membrane M5) measured against a Ag/AgCl wire in 10 mM KCl (1.5 mL) before and after inserting an unconditioned reference membrane doped with [THA^+^]­[C_1_C_1_N^–^] (membrane M1) or [TBMOEP^+^]­[C_1_C_1_N^–^] (membrane M4) into the sample.

## Conclusions

Reference electrodes based on organic electrolytes have recently attracted significant attention due to their versatility and ease of miniaturization. Yet, their transformation into widely adopted commercial tools has been somewhat hindered by the design complications stemming from poor reference membrane lifetimes and their incompatibility with polymeric ISEs, stemming from the excessive transfer of the commonly used moderately lipophilic salts into the aqueous sample. Herein we demonstrated that rational design of the reference electrolyte through controlled lipophilicity tuning of its constituent ions provides a fundamental strategy to overcome these limitations. Ionic liquids based on the bis­(trifluoromethanesulfonyl)­imide anion and a series of tetraalkylammonium cations were synthesized *in-house* via potentiometric titrations to investigate a broad range of organic electrolyte lipophilicity, corresponding to logarithmic partition constants spanning 8.6 ([THA^+^]­[C_1_C_1_N^–^]), 10.6 ([THeptA^+^]­[C_1_C_1_N^–^]) and 12.6 ([TOA^+^]­[C_1_C_1_N^–^]). While reference electrodes based on a PVC/NPDE 1:1 matrix doped with [TOA^+^]­[C_1_C_1_N^–^] performed poorly, indicating that the logarithmic partition constant of 12.6 falls outside the practical lipophilicity limit, [THeptA^+^]­[C_1_C_1_N^–^] and [THA^+^]­[C_1_C_1_N^–^] provided stable electrode potentials. Particularly, reference electrodes incorporating the latter performed on par with previously reported counterparts and exhibited excellent potential stability in a wide range of samples including blood electrolytes, with potential variation of 0.16 mV for solutions spanning the physiological electrolyte range. Importantly, such performance no longer relies on transferring a significant fraction of the ionic liquid into the sample, as the identified optimal lipophilicity range (logarithmic partition constant ≈ 8–9) exceeds that of conventional reference electrolytes by approximately 4 orders of magnitude (logarithmic partition constant ≈ 4–5). Moreover, this improvement is achieved without further complicating the reference membrane composition or relying on expensive membrane components, as required in our previous ionophore-based RE design. The increased lipophilicity reduces the equilibrium aqueous reference electrolyte concentration from the millimolar down to the micromolar range, enabling polymeric ISE-based measurements in small sample volumes for the first time. Although the poorer performance of reference electrodes based on [THeptA^+^]­[C_1_C_1_N^–^] and [TOA^+^]­[C_1_C_1_N^–^] and the nitrate interference observed with [THA^+^]­[C_1_C_1_N^–^] emphasize the need to not only optimize the lipophilicity of the reference electrolyte as a whole but also the lipophilicity balance between its constituent ions, the present work demonstrates that medium-lipophilicity organic electrolytes, rather than commercially available options, may provide the necessary push toward more practical and robust liquid junction-free reference electrodes.

## Supplementary Material



## Data Availability

The original data for this publication are available in Excel format at https://doi.org/10/hb9sr5.

## Notes

Details on estimation of partition constants, additional calibration curves, potential-time traces for temperature-dependent experiments, as well as control experiments (PDF)
