# Supplementary material for: Optimizing Salt Lipophilicity for Reference Electrodes Based on Organic Electrolytes
Source: ACS Meas Sci Au. 2026 Aug 3;6(5):1678–88. doi: 10.1021/acsmeasuresciau.6c00145 (PMC13693250; doi:10.1021/acsmeasuresciau.6c00145)
Supplement: Supplementary file 1 [file tg6c00145_si_001.pdf]

Supporting Information for

Optimizing Salt Lipophilicity for Reference Electrodes Based on Organic Electrolytes

Nikolai Yu. Tiuftiakov [a], Supakron Kittikomoldej [a,b] and Eric Bakker [a]

[a] *Department of Inorganic and Analytical Chemistry, University of Geneva, Quai Ernest-Ansermet 30, CH-1211 Geneva, Switzerland*

[b] *Department of Chemistry and Center of Excellence for Innovation in Chemistry, Faculty of Science, Mahidol University, Rama 6 Road, Bangkok, 10400, Thailand*

Table of Contents

Estimation of ionic liquid partition constants S3
Additional data acquired with $[THA^+][C_1C_1N^-]$-based REs S4
References S7

Estimation of ionic liquid partition constants

The partition constants of various electrolytes were estimated using the partition constant of $[C_8mim^+][C_1C_1N^-]$ ($\log K_{[C_8mim^+][C_1C_1N^-]}$ = 3.6), the ion-exchange constant for tetrabutylammonium [TBA$^+$] and $[C_8mim^+]$ ($\log K_{[TBA^+]/[C_8mim^+]}$ = 1.04), as well as the experimental methylene group lipophilicity increment for the tetraalkylammonium cation series (logarithmic partition constant increases by 0.5 with the addition of one methylene group to the side chain) reported previously for the identical membrane matrix (PVC/dodecyl 2-nitrophenyl ether 1:1). [1] These parameters were incorporated into the following equations (Eq. S1–S4):

$$K_{ij} = k_i\, k_j \tag{S1}$$

$$K_{i/j} = \frac{k_i}{k_j} \tag{S2}$$

$$K_{[Q^+]/[TBA^+]} = \frac{k_{[Q^+]}}{k_{[TBA^+]}} = 2n \tag{S3}$$

$$\log K_{[Q^+][C_8mim^+]} = \log K_{[C_8mim^+][C_1C_1N^-]} + \log K_{\frac{[TBA^+]}{[C_8mim^+]}} + \log K_{\frac{[Q^+]}{[TBA^+]}} \tag{S4}$$

where K_{ij} is a partition constant of an electrolyte *ij*, $K_{i/j}$ is a constant for ion-exchange between ions *i* and *j*, k_i is a single ion partition coefficient of an ion *i*, [Q^+] is the cation of the synthesized IL, n is the side chain length difference between [Q^+] and [TBA$^+$].

Additional data acquired with $[THA^+][C_1C_1N^-]$-based REs

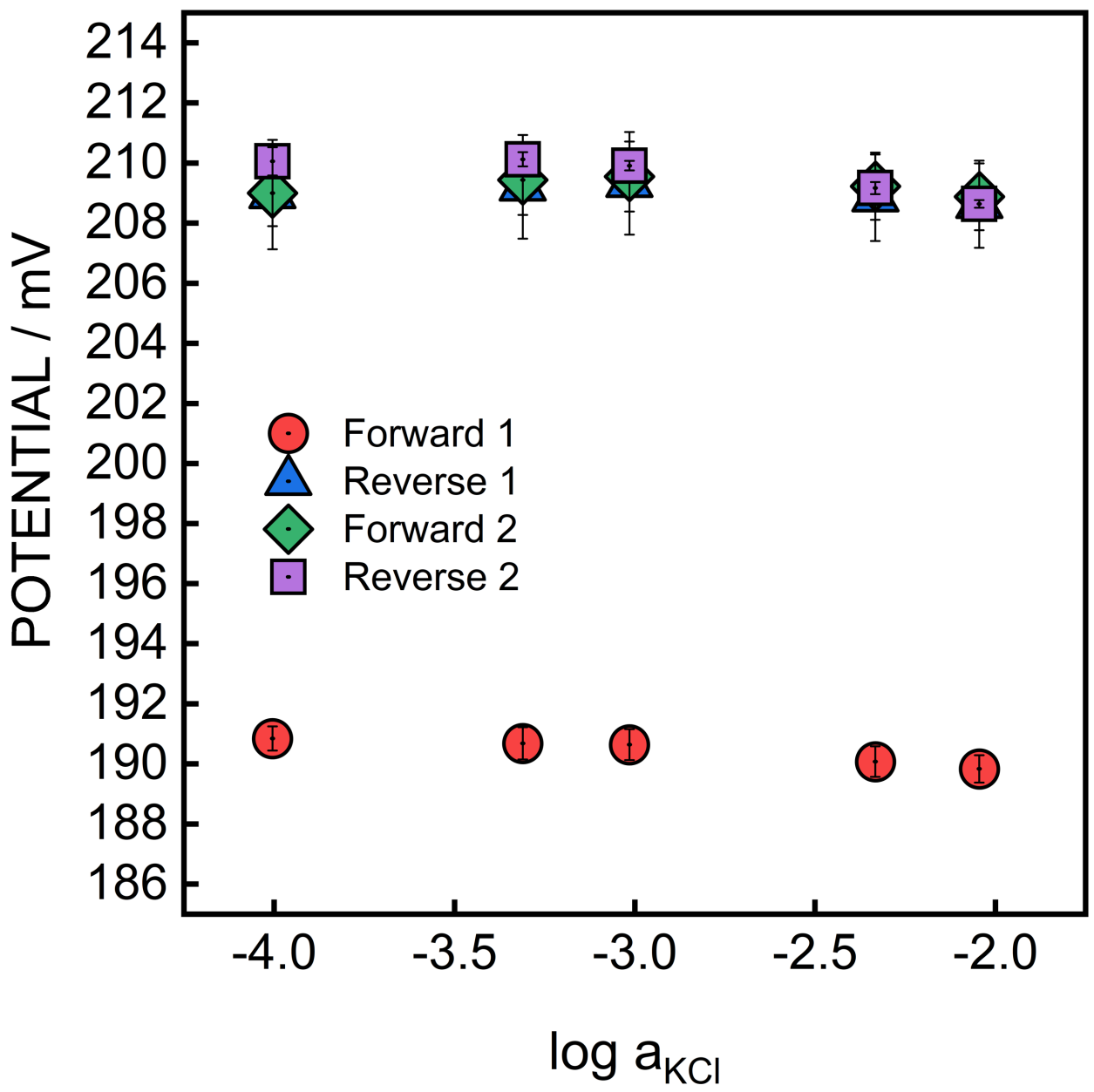

Figure S1. Calibrations of a liquid-contact RE based on $[THA^+][C_1C_1N^-]$ (membrane M1) in KCl solutions while flipping the membrane and renewing the internal filling solution. "Forward" and "Reverse" denote the original and flipped membrane orientations in the electrode body, respectively. Shape center dots represent the measured emf, error bars are standard deviations (n = 3) acquired across three consecutive calibration replicates with the same electrode.

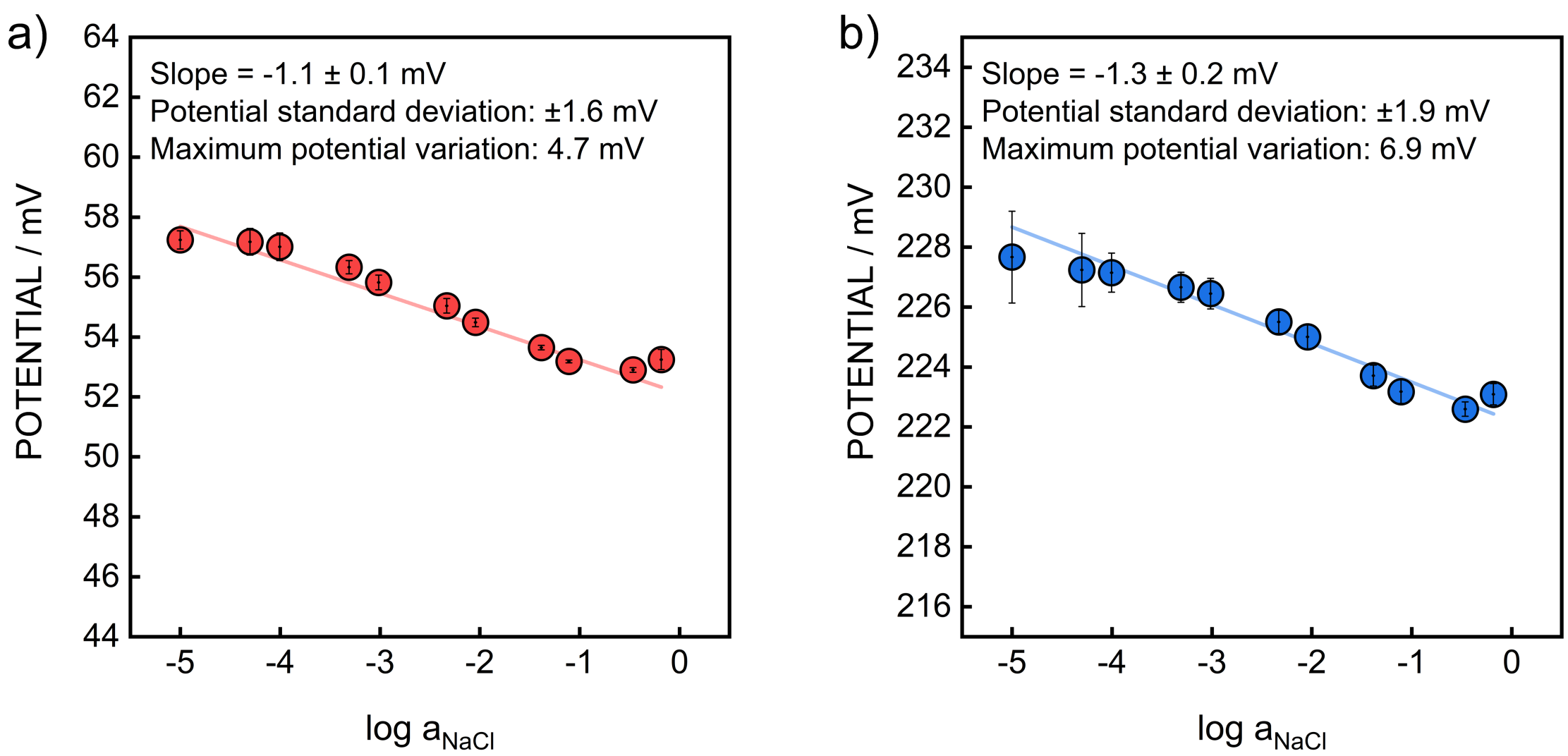

Figure S2. The response of (a) liquid-contact and (b) solid-contact $[THA^+][C_1C_1N^-]$-based REs (membrane M1) in NaCl solutions. Liquid-contact REs were filled with and conditioned in 1 M NaCl prior to the measurements. Circle center dots represent the measured emf, error bars are standard deviations (n = 3) acquired across three consecutive calibration replicates with the same electrode.

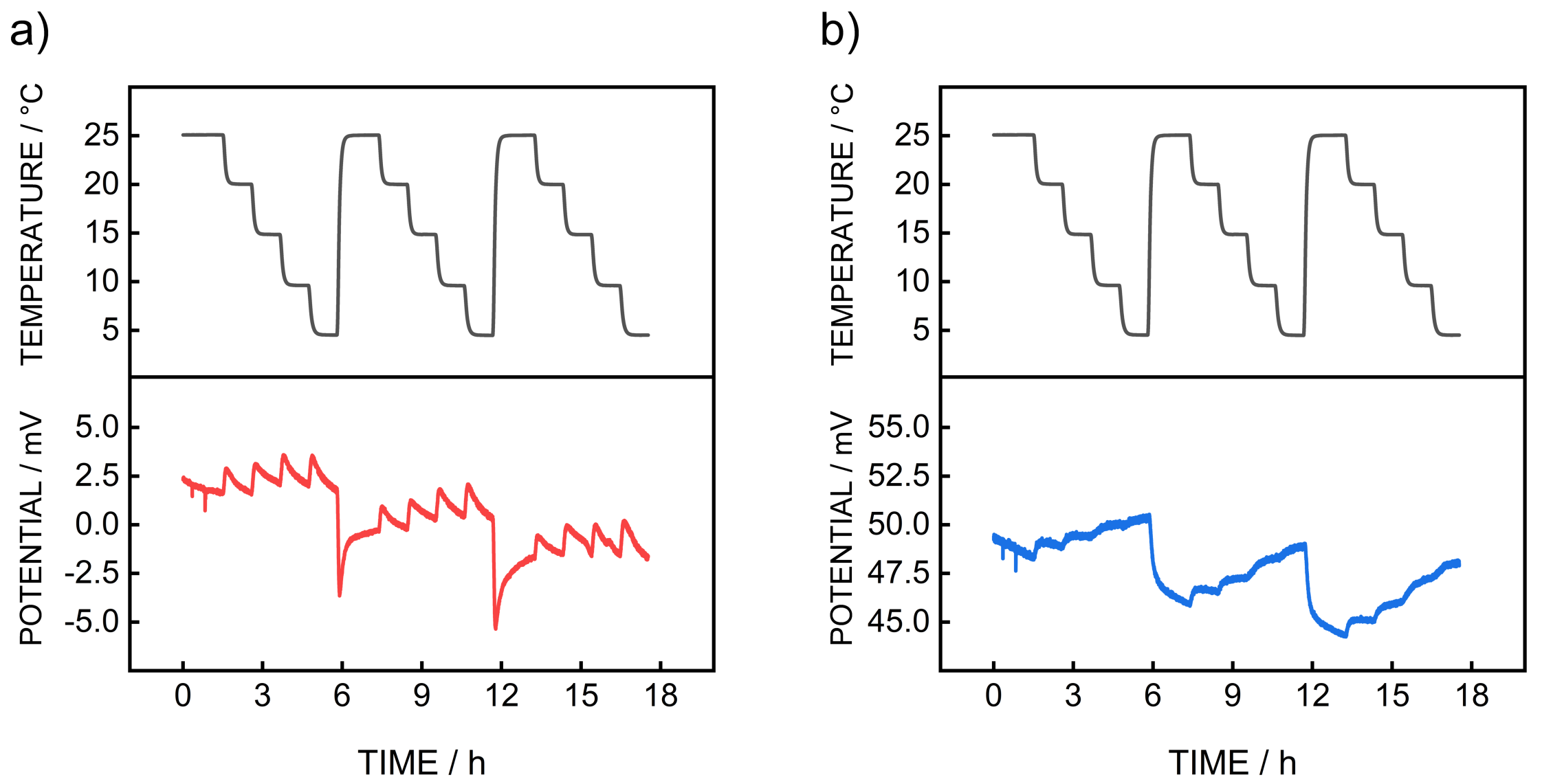

Figure S3. Potential traces of (a) liquid-contact and (b) solid-contact [THA^+][$C_1C_1N^-$]-based REs recorded in a thermostated cell against an Ag/AgCl wire reference electrode in 1 mM KCl.

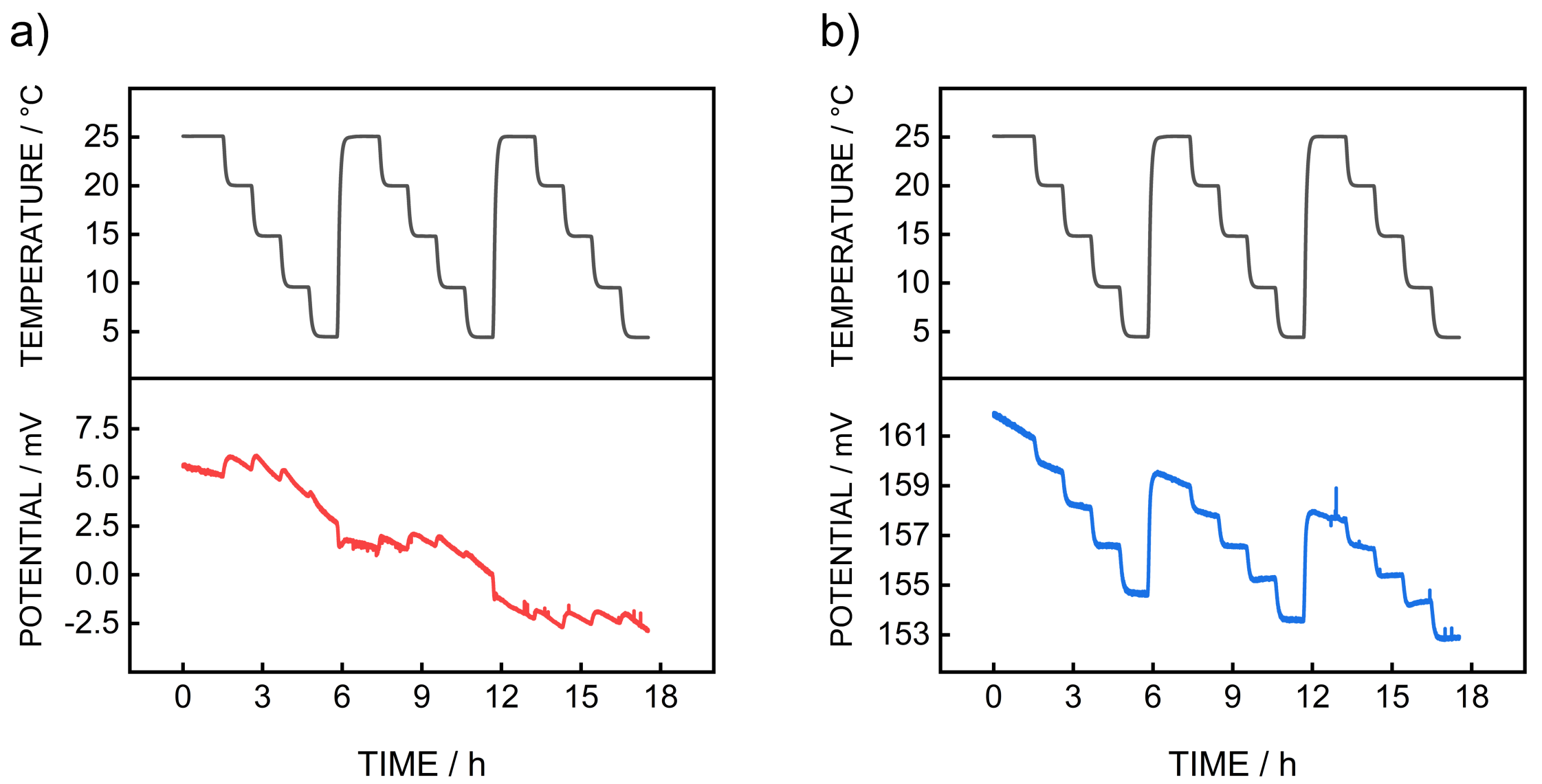

Figure S4. Potential traces of (a) liquid-contact and (b) solid-contact [THA^+][$C_1C_1N^-$]-based REs recorded in a thermostated cell against an Ag/AgCl wire reference electrode in an artificial blood electrolyte (Table 3, "HIGH"). Prior to the measurements, the liquid-contact REs were filled with and conditioned in 0.1 M KCl to establish a symmetrical environment for the internal and external Ag/AgCl elements, thereby compensating for their contribution to the temperature-dependent emf changes of the cell.

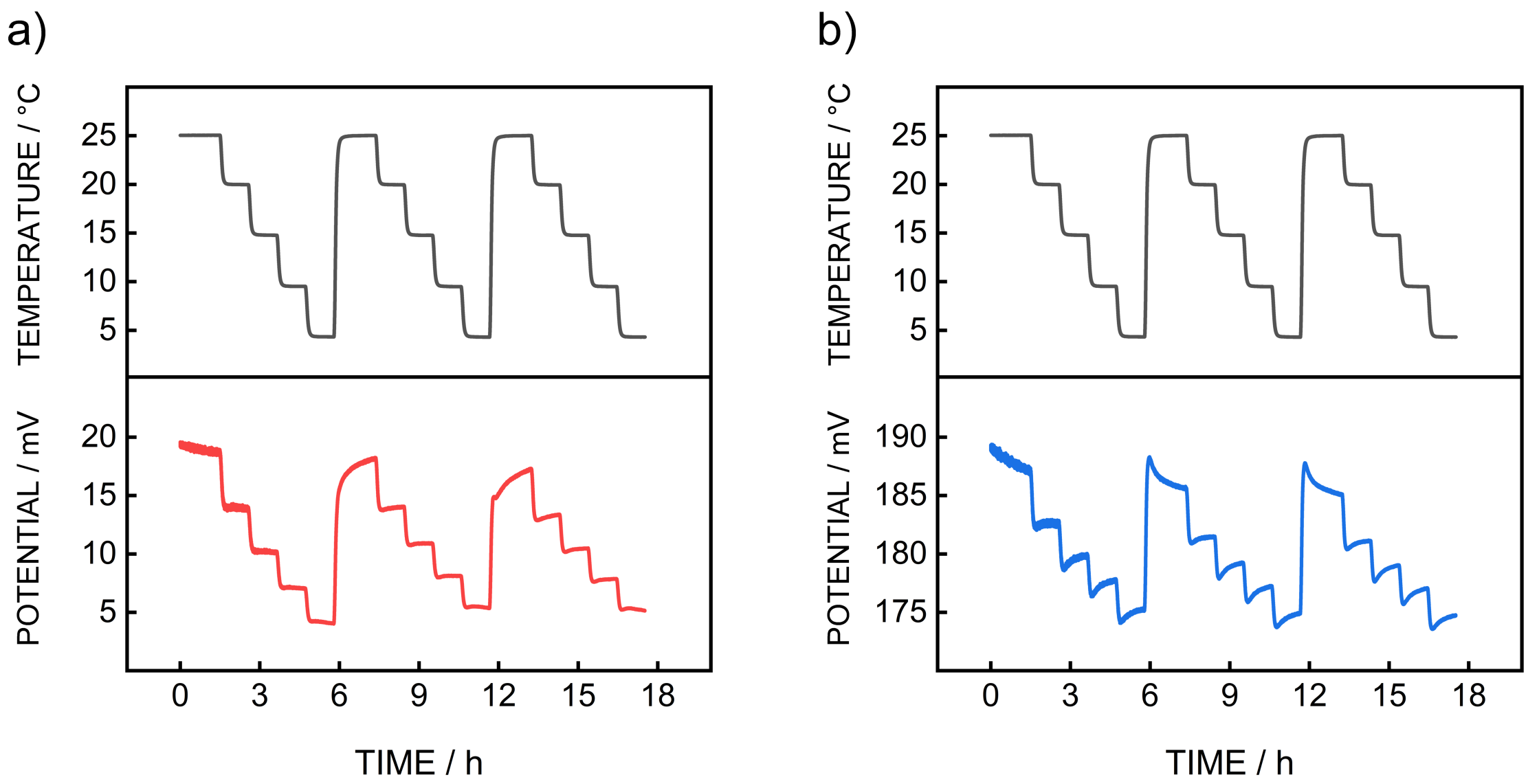

Figure S5. Potential trace of (a) liquid-contact and (b) solid-contact [THA$^+$][$C_1C_1N^-$]-based REs recorded in a thermostated cell against a Ag/AgCl/1 M KCl/3 M KCl reference electrode in 1 mM NaCl. Prior to the measurements, the liquid-contact REs were filled with and conditioned in 1 M NaCl to establish a symmetrical environment for the Ag/AgCl elements of both the liquid junction-free REs and the commercial RE, thereby compensating for their contribution to the temperature-dependent emf changes of the cell.

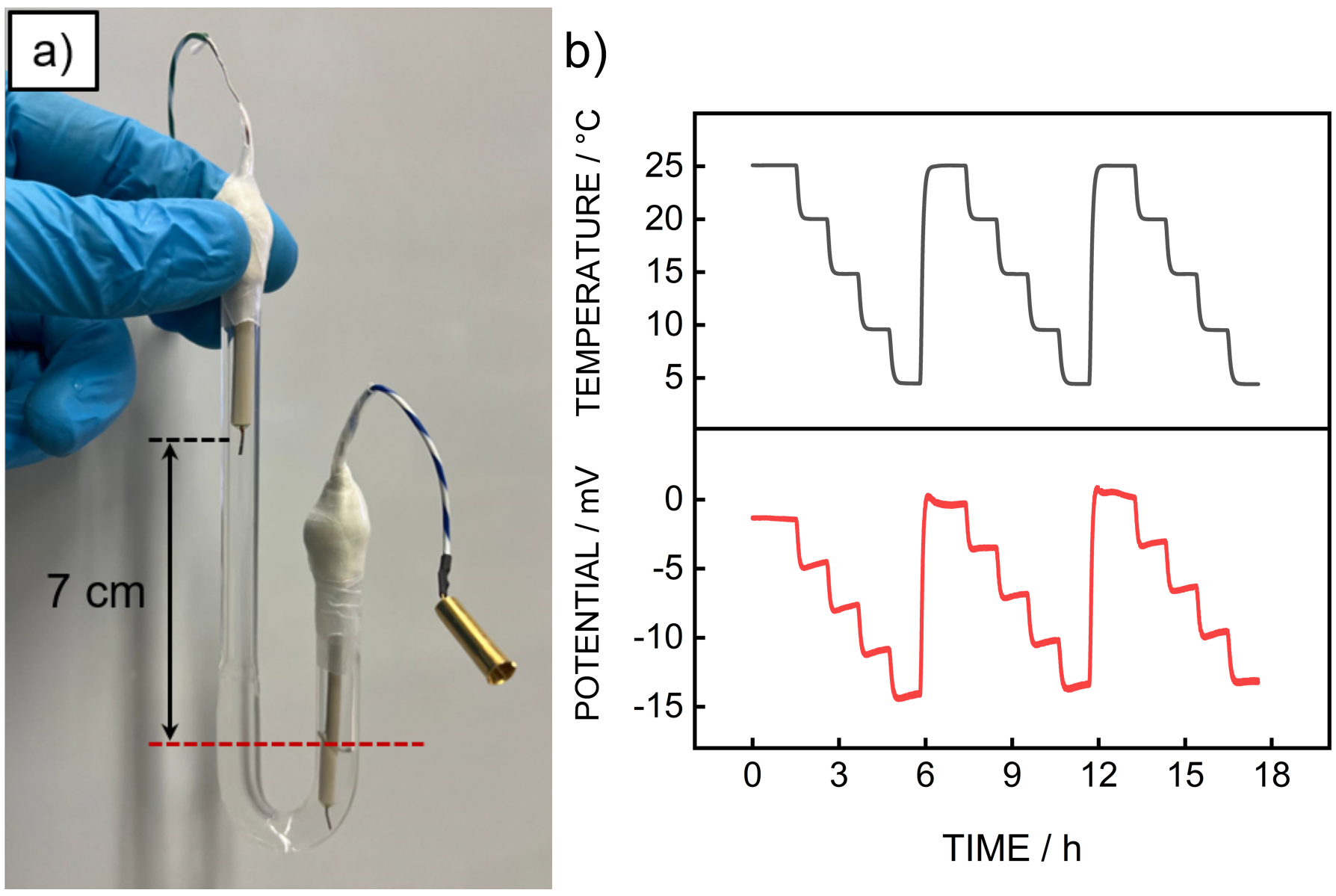

Figure S6. (a) Symmetrical electrochemical cell containing two Ag/AgCl elements immersed in 1 mM KCl used to evaluate the effect of thermal gradients on the results of a temperature ramp experiment, and (b) the corresponding potential–time trace. The red dashed line indicates the immersion depth of the glass tube into the thermostated water bath throughout the experiment.

References

(1) Tiuftiakov, N. Yu.; Zdrachek, E.; Bakker, E. Ion-Exchange and Lipophilicity Limitations of Ionic Liquid Reference Electrodes. *Sens. Actuators B Chem.* **2024**, *407*, 135474. https://doi.org/10.1016/j.snb.2024.135474.
